# Regional, demographic and temporal trends in anemia and malignant cancer-related mortality in U.S. older adults: a nationwide CDC WONDER analysis (1999–2020)

**DOI:** 10.3389/fonc.2026.1722891

**Published:** 2026-01-21

**Authors:** Muhammad Sarim Azad Khan, Asma Chaudhary, Mirha Imran Khan, Ibrahiem Azeem Ajaz, Arham Khalid, Aroosha Waheed, Mohammed Hammad Jaber Amin

**Affiliations:** 1 Department of Medicine, Combined Military Hospital Lahore Medical College, Lahore, Pakistan; 2 Department of Medicine, Fazaia Medical College, Islamabad, Pakistan; 3 Department of Medicine, Combined Military Hospital Institute of Medical Science, Multan, Pakistan; 4 Department of Medicine, Rashid Latif Medical College, Lahore, Pakistan; 5 Department of Medicine, Rawalpindi Medical University, Rawalpindi, Pakistan; 6 Department of Medicine, Alzaiem Alazhari University, Khartoum, Sudan

**Keywords:** age-adjusted mortality rate, anemia, cancer, CDC WONDER, epidemiology, mortality trends

## Abstract

**Purpose:**

Anemia and malignancy concurrently contribute to reduced quality of life, treatment intolerance, and increased morbidity and mortality, disproportionately affecting older adults, socioeconomically disadvantaged groups, and minority populations. Despite its clinical importance, national patterns and population-level disparities in the mortality burden associated with this comorbidity remain poorly characterized. This study aims to examine mortality trends associated with concurrent anemia and malignancy in the United States using CDC WONDER (Centers for Disease Control and Prevention Wide-Ranging Online Data for Epidemiologic Research) database, with a focus on demographic and geographic disparities to inform targeted interventions.

**Methods:**

Mortality data from CDC WONDER were analyzed for individuals aged 65 years and older using ICD-10 codes C00–C97 for malignancies and D50–D64 for anemia. Age-adjusted mortality rates (AAMRs) and annual percent changes (APCs) were calculated by year, age, sex, race/ethnicity, cancer type, and geographic region. Subgroup analyses focused on common anemia-associated cancers, including colorectal, gastric, lung, ovarian, female genital, breast, prostate, and hematologic malignancies, based on national prevalence.

**Results:**

Between 1999 and 2020, there were 264,331 anemia and malignancy -related deaths among U.S. adults aged ≥65. The overall AAMR remained relatively stable moving from 31.5 in 1999 to 31.1 in 2020. A significant decline occurred through 2017 (APC: –0.86), followed by a sharp increase from 2017 to 2020 (APC: 4.63). Males had higher AAMRs than females (28.8 vs. 22.6). Black individuals had the highest mortality (42.4). The Midwest had the highest regional AAMR (30.6), while the South had the lowest (27.6). States in the top 90th percentile for AAMR included North Dakota, Maryland, Rhode Island, West Virginia, and DC. Nonmetropolitan areas had higher AAMRs than metropolitan ones (32.9 vs. 27.8). Colorectal cancer showed the largest decline (AAPC: -2.03) followed by gastric (AAPC: -1.96) and prostate cancer (AAPC: -1.40), while breast, lung, gynecological, and hematological cancers remained stable.

**Conclusion:**

Among adults aged ≥65, anemia and malignancy-related mortality declined until 2017 but increased sharply through 2020, with highest rates in males, Black individuals, and nonmetropolitan/Midwestern residents. Declines were seen in colorectal, gastric, and prostate cancers, while other cancers remained stable, highlighting the need for targeted interventions to reduce disparities.

## Highlights

- Concurrent anemia and malignancy related mortality declined from 1999–2017 but rose significantly from 2017–2020.
- NH Black individuals had the highest AAMR
- Males and nonmetropolitan residents showed highest mortality rates.
- The Midwest and certain states (WV, MD, ND) had the highest mortality burdens.
- Colorectal cancer demonstrated the steepest decline [AAPC: -2.03] followed by gastric cancer [AAPC: -1.96] and prostate cancer [AAPC: -1.40]
- Lung cancer exhibited an initial increase from 1999 to 2012, a period of stability from 2012 to 2015 and a subsequent sharp rise from 2015 to 2020.

## 1 Introduction

Cancer remains a major public health challenge in the United States, with over 1.6 million new cases annually (1). Despite advances in detection and treatment, it is projected to surpass cardiovascular disease as the leading cause of death (2). Anemia, the third leading global cause of years lived with disability (YLD) (3), contributed to 5,928 deaths in 2023, with a mortality rate of 1.8 per 100,000 population (4). The etiology of cancer-related anemia (CRA) is multifactorial, involving bone marrow infiltration, cytokine-induced iron sequestration, shortened red cell survival, chronic blood loss, oxidative stress and organ dysfunction (5, 6). Both severe anemia and CRA often necessitates repeated blood transfusions, which are life-saving in the acute setting but can lead to long-term complications such as iron overload, organ toxicity, alloimmunization, and transfusion reactions that complicate future management (7, 8). Over time, these sequelae contributes to poorer clinical outcomes and increased mortality among affected patients (9). Beyond its clinical toll, anemia in the setting of malignancy imposes substantial health and economic burdens (10).

Nephrotoxic agents such as cisplatin reduce erythropoietin production, while newer treatments, including antibody–drug conjugates, tyrosine kinase inhibitors, PARP inhibitors, and immunotherapies, can impair erythropoiesis, promote inflammatory pathways and worsen existing anemia, further complicating management (11–13). The European Cancer Anemia Survey (ECAS) reported that nearly 40% of chemotherapy patients had hemoglobin levels below 10 g/dL, underscoring the high prevalence and ongoing management challenges associated with anemia in oncology (12).

Anemia prevalence varies widely across cancer types, affecting approximately 40–50% of patients with prostate cancer, 36% with hematologic malignancies, 30% with breast cancer (BC), 23% with lung cancer, and 25% with colorectal cancer (CRC), with rates increasing with disease progression (14, 15). Although the prognostic significance of anemia remains debated, evidence consistently associates it with poorer functional status, reduced treatment tolerance, and increased mortality across several malignancies (16). Socioeconomic and demographic disparities further exacerbate these outcomes, with this comorbidity disproportionately affecting socioeconomically deprived and rural populations, as well as Black, Hispanic, and American Indian/Alaska Native individuals, despite lower cancer incidence rates (17, 18). In older adults (>65 years), anemia is an established risk factor for hospitalization, postoperative complications, and higher all-cause mortality (19, 20).

However, most existing research has focused on clinical outcomes within specific cancer subtypes or treatment settings (21–23), leaving substantial uncertainty regarding national patterns and population-level disparities in mortality associated with anemia and malignancy, especially in older adults. A comprehensive evaluation across demographic and geographic subgroups is crucial to better quantify the public health burden and inform equitable cancer care strategies. To address this gap, the present study utilizes the CDC WONDER (Centers for Disease Control and Prevention Wide-Ranging OnLine Data for Epidemiologic Research) database to assess national trends in concurrent anemia and malignancy mortality in the United States. The findings aim to offer an epidemiologic foundation for evidence-based interventions and policy initiatives to reduce disparities in cancer outcomes.

## 2 Methodology

### 2.1 Study setting and population

The CDC WONDER database was utilized to extract death certificate data spanning from 1999 to 2020, covering all 50 U.S. states and the District of Columbia. The dataset was accessed on October 3^rd^ 2025 (24). Our analysis utilized the ‘Multiple Cause of Death’ Public Use dataset, including all cases in which anemia and malignant neoplasms were recorded as either the underlying or a contributing cause of death (ie, listed anywhere on the death certificate). Malignant neoplasms were identified using ICD-10 codes C00–C97, while anemia was classified using codes D50–D53 (Nutritional anemias), D55-D59 (Hemolytic anemias), and D60-D64 (Aplastic and other anemias). Subgroup analyses utilized ICD-10 codes to identify specific malignancy types listed in the Multiple Cause of Death fields. Anemia (D50–D64) was a mandatory contributing cause for inclusion in all subtype-specific mortality calculations. To capture the comprehensive mortality burden involving these organ systems, our coding definitions included both primary neoplasms and where relevant, secondary (metastatic) involvement. Specifically, we defined the subgroups as: CRC (C18–C20, C21.8, C78.5); Gastric Cancer (C16); Lung Cancer (C34, C78.0); BC (C50); Prostate Cancer (C61); Gynecologic Cancers (C51–C58, C79.6); and Hematologic Malignancies (C81–C96). According to the latest national statistics from the United States, these cancer types were chosen to reflect the most prevalent cancers causing anemia. These same ICD-10 codes have been validated and employed in previous studies to categorize patients with malignancies and anemia (22, 25). Under this inclusive “Multiple Cause” framework, a single death certificate listing multiple distinct malignancies (e.g., primary BC with secondary lung involvement) contributed to the mortality rates of both respective subgroups, reflecting the complex comorbidity profile of the decedent. Hematologic malignancies were aggregated into a composite category encompassing lymphomas, multiple myeloma, and all acute/chronic leukemias (C81–C96) to assess the collective burden of hematopoeitic neoplasms. In compliance with CDC WONDER data confidentiality policies, mortality counts between one and nine were suppressed. Consequently, specific demographic or geographic stratifications yielding suppressed counts were deemed unreliable and excluded from the final analysis to ensure statistical robustness. This study follows STROBE (Strengthening the Reporting of Observational Studies in Epidemiology) guidelines (26).

### 2.2 Data abstraction

For this study, data were extracted on multiple mortality-related variables, including gender, year of death, state of residence, place of death, age distribution (≥65), urban–rural status, and regional categorization. Demographic factors comprised age and race/ethnicity, with race/ethnicity classified as non-Hispanic (NH) White, NH Black or African American, Hispanic or Latino, NH American Indian or Alaska Native, and NH Asian or Pacific Islander. These race/ethnicity categories have previously been used within analyses from the CDC WONDER database and rely on reported data on death certificates. Place of death was grouped into medical facilities (outpatient, emergency department, inpatient, death on arrival, or unspecified), home, hospice, or nursing home/long-term care settings. Urban–rural status was assigned using the 2013 National Center for Health Statistics classification, defining counties as urban (large metropolitan ≥1 million; medium/small metropolitan 50,000–999,999) or rural (<50,000) (27). Geographic regions followed U.S. Census Bureau definitions: Northeast, Midwest, South, and West (28).

### 2.3 Data analysis

To analyze national trends in anemia and malignancy-related mortality, crude rates (CMR) and age-adjusted mortality rates (AAMRs) per 100,000 population were calculated for the years 1999 to 2020, along with their corresponding 95% confidence intervals (CIs). CMRs were determined by dividing the number of concurrent anemia and malignant neoplasms-related deaths by the corresponding United States population of that year. AAMRs were calculated by standardizing the anemia and malignant neoplasm-related deaths to the 2000 United States population as previously described (29). These rates were stratified by sex, race/ethnicity, state, urban-rural classification, census region, metropolitan status, and year. Annual percent changes (APCs) and 95% CIs in AAMRs were estimated using the Joinpoint Regression Program (Version 5.3.0, National Cancer Institute) to assess trends and detect significant inflection points over time (30). This approach detects significant temporal shifts in AAMRs by applying log-linear regression models to periods of change. APCs and their 95% CIs were derived for each segment between joinpoints using the Monte Carlo permutation test to identify the optimal number of inflection points, conducting 4,499 permutations. A maximum of four joinpoints was permitted. Trends were classified as increasing or decreasing when the slope of mortality change significantly differed from zero. A two-tailed *t*-test was used to determine the statistical significance of APCs, with a *P*-value less than 0.05 considered statistically significant. Models were fit using the grid-search method assuming constant variance and uncorrelated errors. Population denominators were not smoothed. For stratified analyses (e.g., by sex or race), identical algorithm settings were applied, but models were fit independently without ensuring parallel trends or identical joinpoint locations, allowing the software to detect distinct temporal patterns for each demographic group.

## 3 Results

Between 1999 and 2020, there were 264,331 anemia and malignancy related deaths among U.S. adults aged 65 and older (**Table 1**). These deaths occurred across various settings, with the majority taking place in medical facilities (38.19%), followed by deaths at home (27.88%), in nursing homes or long-term care facilities (25.49%), hospice settings (4.37%), and other locations (3.88%) (**Supplementary Table 1**).

**Table 1:** Anemia and malignancy–related deaths, stratified by sex and race in older adults in the United States, 1999 to 2020.

Year	Overall	Women	Men	Hispanic/Latino	NH American indian or Alaska native	NH Asian or Pacific Islander	NH black or African American	NH White	Population
1999	10863	5267	5596	389	25	175	1466	8781	34797841
2000	10717	5222	5495	372	30	194	1480	8623	34991753
2001	10746	5319	5427	407	35	196	1423	8659	35290291
2002	10876	5259	5617	445	41	199	1439	8727	35522207
2003	10839	5244	5595	431	47	215	1416	8707	35863529
2004	10836	5227	5609	444	35	253	1379	8700	36203319
2005	10746	5217	5529	513	37	232	1301	8647	36649798
2006	11072	5381	5691	520	45	243	1337	8912	37164107
2007	10976	5248	5728	559	45	267	1390	8705	37825711
2008	11356	5474	5882	529	46	295	1398	9066	38777621
2009	11117	5297	5820	568	44	312	1338	8832	39623175
2010	11883	5565	6318	620	69	346	1403	9432	40267984
2011	11660	5438	6222	651	58	340	1384	9222	41394141
2012	12146	5646	6500	780	63	364	1411	9499	43145356
2013	12287	5736	6551	802	63	345	1396	9657	44704074
2014	12372	5760	6612	789	62	406	1494	9596	46243211
2015	12334	5609	6725	823	50	400	1426	9594	47760852
2016	12653	5772	6881	888	53	353	1446	9885	49244195
2017	13463	6128	7335	945	74	460	1605	10348	50858679
2018	14104	6458	7646	966	64	469	1603	10980	52431193
2019	14694	6662	8032	1033	87	506	1636	11404	54058263
2020	16591	7491	9100	1128	94	519	2016	12808	55659365
total	264331	124420	139911	14602	1167	7089	32187	208784	928476665

### 3.1 Annual trends in AAMR for anemia and malignancy

**Figure 1:**
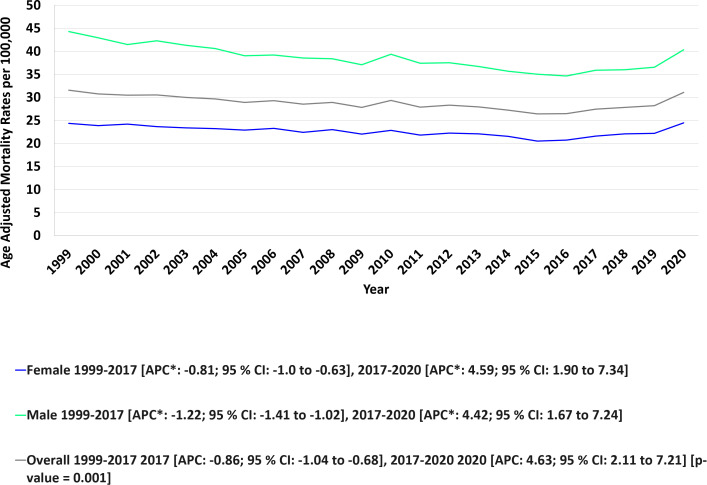
Overall and sex stratified anemia and malignancy related age-adjusted mortality rates per 100,000 among older adults aged ≥65 years in the United States, 1999–2020.*Indicates that the annual percent change (APC) is significantly different from zero at α = 0.05. AAMR, age-adjusted mortality rate; APC, annual percent change; CI, confidence interval.

**Figure 2:**
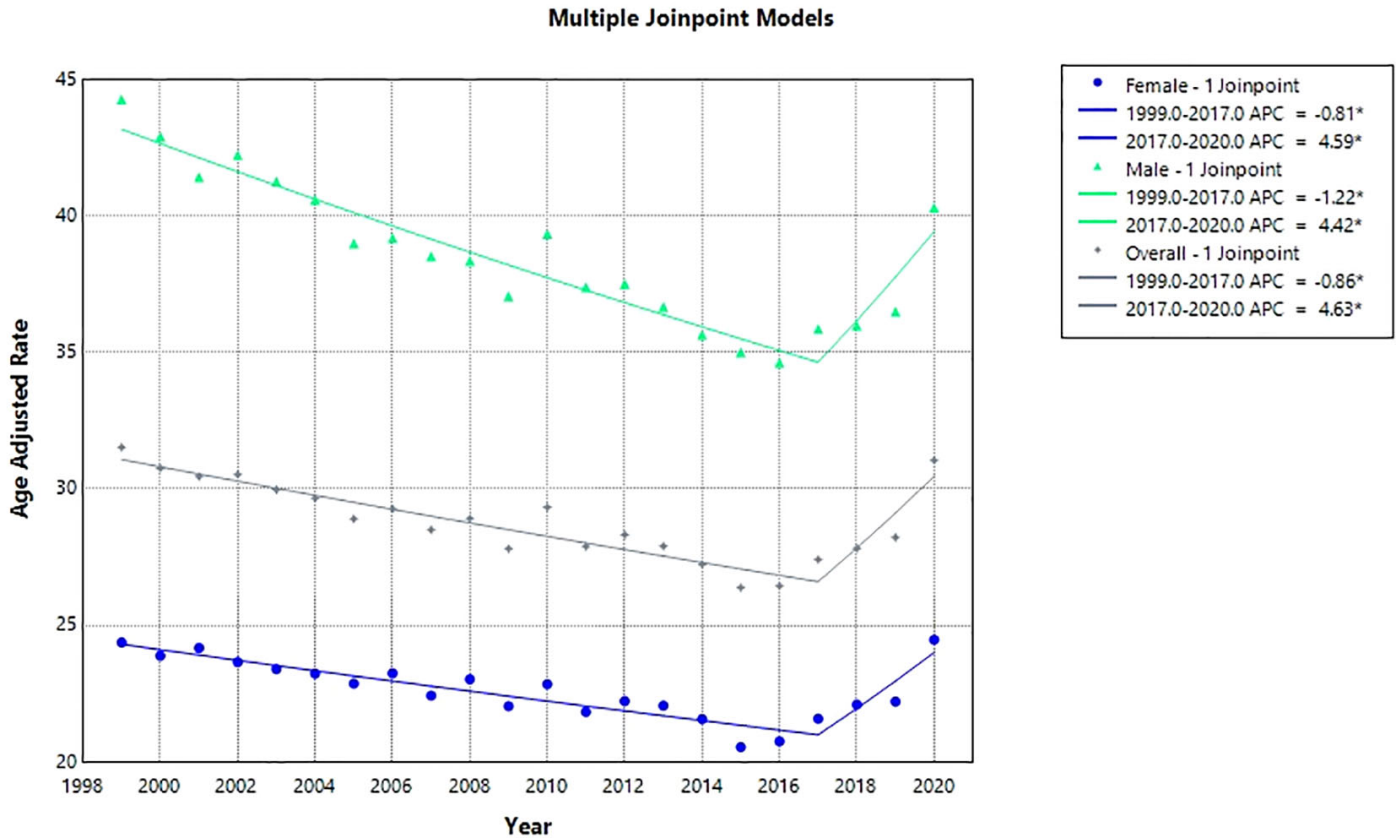
Joinpoint model of anemia and malignancy-related AAMR per 100,000; overall and stratified by sex, 1999-2020 (*indicates the APC is statistically significant).

Over the 22-year study period, the overall AAMR for concurrent anemia and malignancy among older adults remained relatively stable, moving from 31.53 per 100,000 population in 1999 to 31.05 in 2020. Joinpoint analysis revealed a statistically significant downward trend from 1999 through 2017 (APC: –0.86; 95% CI: –1.04 to –0.68), followed by a sharp increase from 2017 through 2020 (APC: 4.63; 95% CI: 2.11 to 7.21).(AAPC: -0.09; 95% CI: -0.45 to 0.26, p value>0.05). (**Figures 1**, **2**, **Tables 2**, **3**).

**Table 2:** Annual percent change (APC) of anemia and malignancy related age-adjusted mortality rates per 100,000 in older adults the United States, 1999 to 2020.

Year	APC (95% CI)
Overall	
1999-2017	-0.86 (-1.04- -0.68)
2017-2020	4.63 (2.12-7.21)
Male	
1999-2017	-1.22 (-1.41- -1.20)
2017-2020	4.42 (1.67-7.24)
Female	
1999-2017	-0.81 (-1.00 - -0.63)
2017-2020	4.59 (1.90-7.34)
NH American Indian or Alaskan Native	
1999-2020	-0.46 (-1.64-0.73)
NH Black or African American	
1999-2018	-2.49 (-2.74- -2.25)
2018-2020	8.04 (0.08-16.63)
Hispanic or Latino	
1999-2020	0.25 (-0.56 -0.06)
NH Asian or Pacific Islander	
1999-2020	-1.50 (-2.03 - -0.97)
Metropolitan Areas	
1999-2017	-0.88 (-1.09 - -0.66)
2017-2020	4.44 (1.49-7.47)
Non Metropolitan Areas	
1999-2016	-0.82 (-1.09- -0.55)
2016-2020	4.06 (1.79-6.39)
Northeast	
**1999-2020**	-0.95 (-1.22 - -0.68)
Midwest	
**1999-2016**	-1.49 (-1.69- -1.28)
**2016-2020**	4.18 (2.35-6.04)
West	
**1999-2018**	-0.48 (-0.80-0.16)
**2018-2020**	5.18 (-3.94-15.17)
South	
**1999-2016**	-0.66 (-0.89- -0.43)
**2016-2020**	4.70 (2.88-6.55)

**Table 3:** Overall and sex‐stratified anemia and malignancy related age-adjusted mortality rates per 100,000 in older adults in the United States, 1999 to 2020.

Year	Male	Female	Overall
1999	44.29 (43.10–45.47)	24.37 (23.71–25.03)	31.53 (30.93–32.12)
2000	42.93 (41.77–44.08)	23.88 (23.23–24.53)	30.76 (30.17–31.34)
2001	41.44 (40.32–42.56)	24.17 (23.52–24.82)	30.46 (29.89–31.04)
2002	42.25 (41.13–43.37)	23.65 (23.01–24.29)	30.53 (29.96–31.11)
2003	41.28 (40.19–42.38)	23.39 (22.76–24.03)	29.97 (29.41–30.54)
2004	40.61 (39.54–41.69)	23.23 (22.60–23.87)	29.66 (29.10–30.22)
2005	39.01 (37.97–40.05)	22.86 (22.24–23.49)	28.90 (28.36–29.45)
2006	39.21 (38.18–40.23)	23.24 (22.62–23.87)	29.27 (28.72–29.81)
2007	38.54 (37.53–39.54)	22.42 (21.80–23.03)	28.50 (27.96–29.03)
2008	38.37 (37.39–39.36)	23.02 (22.40–23.63)	28.91 (28.38–29.44)
2009	37.07 (36.11–38.02)	22.03 (21.43–22.63)	27.81 (27.29–28.33)
2010	39.36 (38.38–40.33)	22.84 (22.23–23.44)	29.33 (28.80–29.85)
2011	37.40 (36.46–38.33)	21.82 (21.23–22.41)	27.89 (27.38–28.39)
2012	37.52 (36.60–38.44)	22.22 (21.63–22.81)	28.32 (27.81–28.82)
2013	36.69 (35.80–37.59)	22.05 (21.47–22.63)	27.90 (27.40–28.39)
2014	35.66 (34.79–36.53)	21.56 (20.99–22.12)	27.23 (26.74–27.71)
2015	35.02 (34.17–35.87)	20.53 (19.98–21.07)	26.38 (25.91–26.85)
2016	34.63 (33.80–35.46)	20.74 (20.20–21.29)	26.44 (25.97–26.90)
2017	35.88 (35.05–36.71)	21.57 (21.03–22.12)	27.41 (26.94–27.88)
2018	35.99 (35.17–36.81)	22.09 (21.55–22.64)	27.82 (27.36–28.29)
2019	36.51 (35.70–37.32)	22.20 (21.66–22.74)	28.22 (27.76–28.68)
2020	40.32 (39.48–41.16)	24.47 (23.91–25.03)	31.05 (30.58–31.53)
total	38.24 (38.04–38.44)	22.61 (22.48–22.74)	28.78 (28.67–28.89)

### 3.2 Anemia and malignancy-related AAMR stratified by gender

Throughout the study period, men consistently had higher overall AAMRs than women (AAMR: 28.78 [95% CI: 28.67–28.89] for men vs. 22.61 [95% CI: 22.48–22.74] for women). Among men, AAMR declined over time (AAPC: –0.43; 95% CI: –0.82 to –0.04), while it remained stable for women (AAPC: –0.06; 95% CI: –0.43 to 0.32; *p* = 0.76).

For men, AAMR fell markedly from 44.29 in 1999 to 35.88 in 2017 (APC: –1.22; 95% CI: –1.41 to –1.02), then rose significantly to 40.99 in 2020 (APC: 4.42; 95% CI: 1.67 to 7.24). For women, the AAMR decreased from 24.37 in 1999 to 21.57 in 2017 (APC: –0.81; 95% CI: –1.00 to –0.63), followed by a significant increase to 24.47 in 2020 (APC: 4.59; 95% CI: 1.90 to 7.34) (**Figures 1**, **2**, **Tables 2**, **3**).

### 3.3 Anemia and malignancy-related AAMR stratified by race/ethnicity

**Figure 3:**
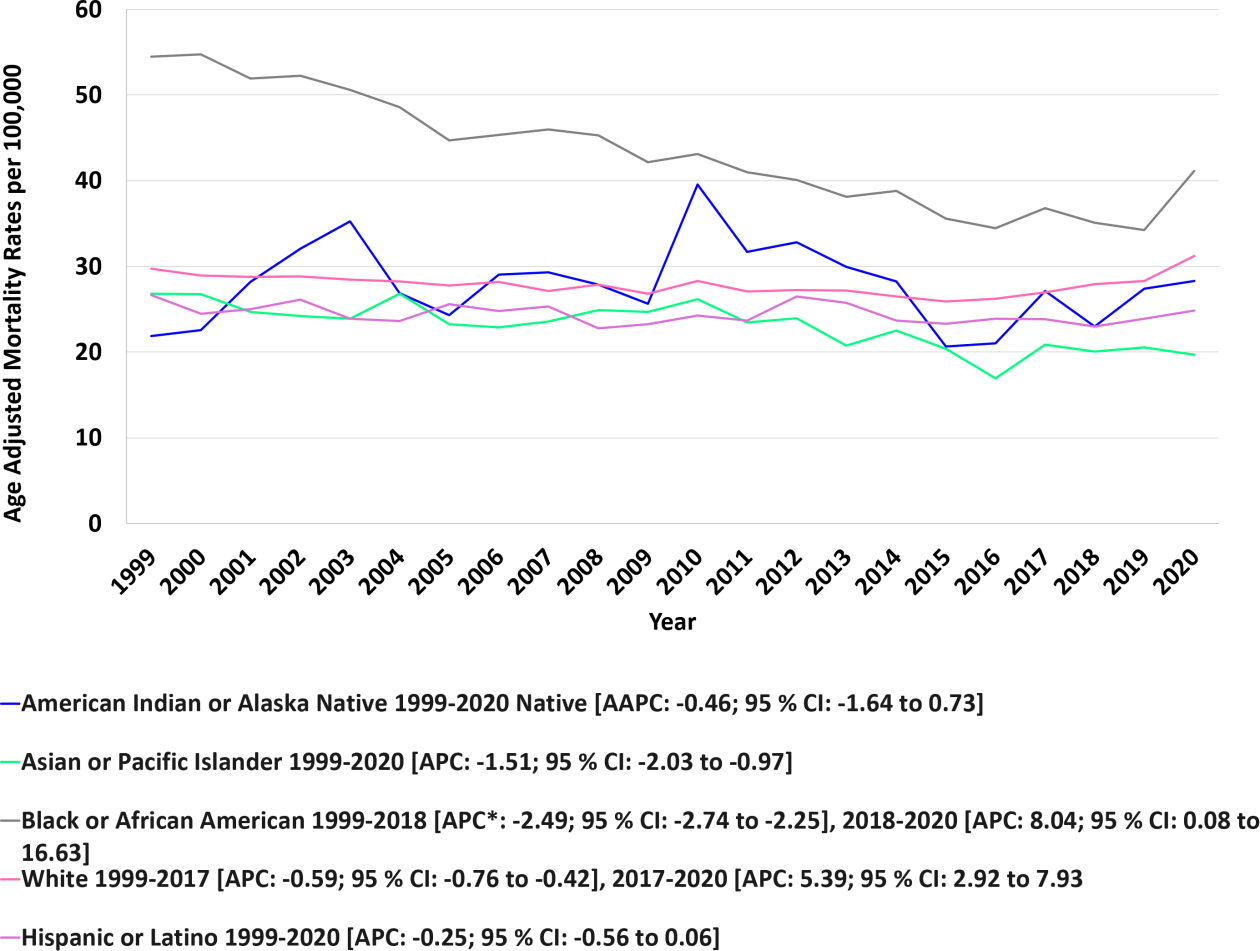
Race stratified anemia and malignancy related age-adjusted mortality rates per 100,000 among older adults aged ≥65 years in the United States, 1999–2020.* Indicates that the annual percent change (APC) is significantly different from zero at α = 0.05. AAMR, age-adjusted mortality rate; APC, annual percent change; CI, confidence interval.

**Figure 4:**
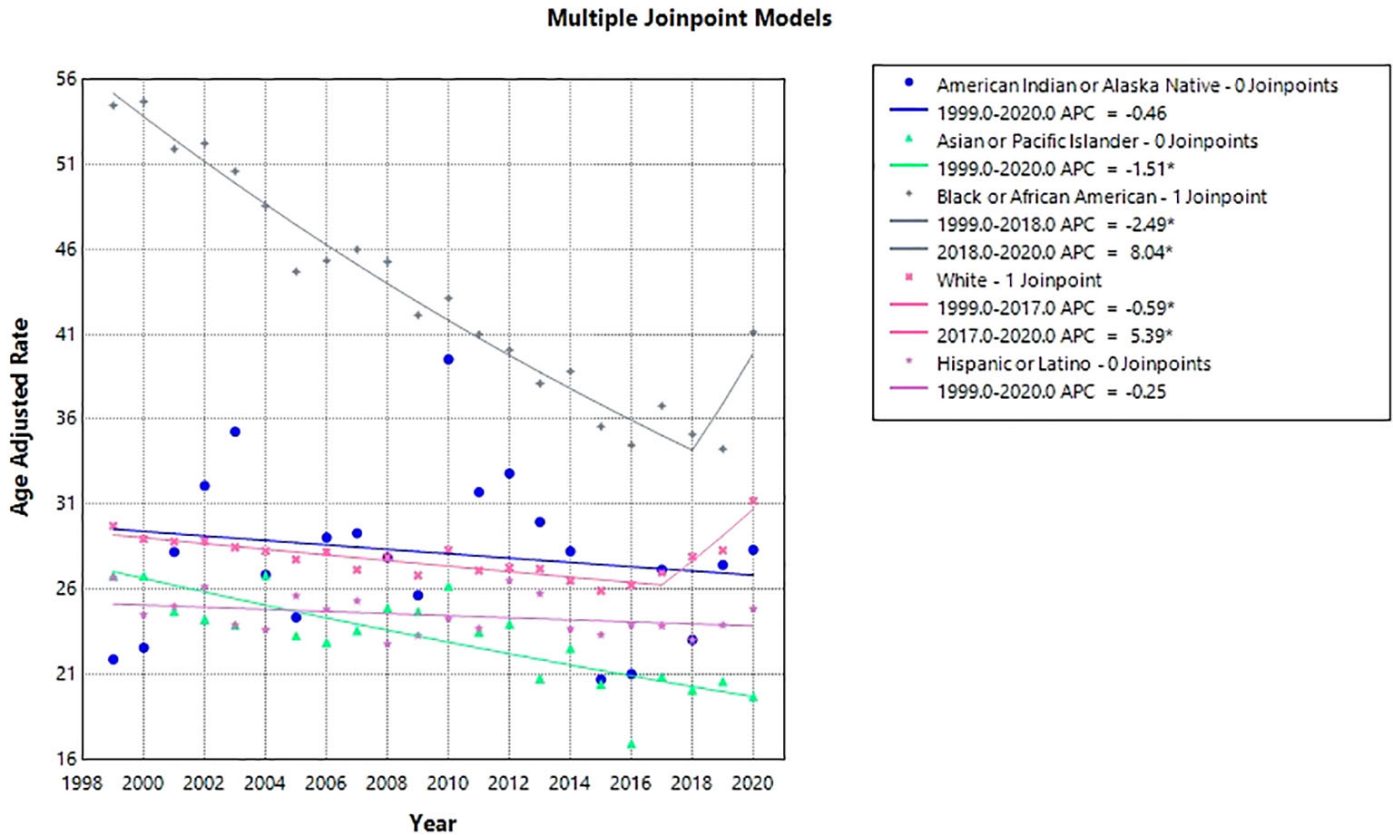
Joinpoint model of anemia and malignancy-related AAMR per 100,000; stratified by race, 1999-2020 (*indicates the APC is statistically significant).

The highest number of anemia and malignancy- related deaths occurred among NH White individuals (208,784), followed by NH Black (32,187), Hispanic (14,602), NH Asian or Pacific Islander (7,089), and NH American Indian or Alaska Native populations (1,167). Overall AAMRs were highest among NH Black or African Americans, followed by NH Whites, NH American Indian or Alaska Natives, Hispanic or Latinos, and NH Asian or Pacific Islanders (NH Black or African American: 42.39, 95% CI: 41.93 to 42.86; NH White: 27.88, 95% CI: 27.76 to 28.00;NH American Indian or Alaska Native: 27.53, 95% CI: 25.91 to 29.15; Hispanic or Latino: 24.25, 95% CI: 23.85 to 24.64; NH Asian or Pacific Islander: 22.16, 95% CI: 21.64 to 22.68). From 1999 to 2020, AAMRs remained relatively stable among NH American Indian or Alaska Native (AAPC: -0.46; 95% CI: -1.64 to 0.73; p-value = 0.43), Hispanic or Latino populations (AAPC: -0.25; 95% CI: -0.56 to 0.06; p-value = 0.10) and NH Whites (AAPC: 0.24; 95% CI: 0.10 to 0.58; p-value=0.16). A significant downward trend was observed in NH Asian or Pacific Islanders (APC: -1.51; 95% CI: -2.03 to -0.97; p-value = 0.00001). Among NH Black or African Americans, AAMR decreased from 1999 through 2018 (APC: -2.49; 95% CI: -2.74 to -2.25; p-value <0.000001), followed by a sharp rise from 2018 to 2020 (APC: 8.04; 95% CI: 0.08 to 16.63; p-value = 0.047). A similar pattern was observed among NH White populations, with a significant decline from 1999 through 2017 (APC −0.59; 95% CI −0.76 to −0.42; *p* < 0.001), followed by a significant increase through 2020 (APC 5.39; 95% CI 2.92 to 7.93; *p* < 0.001) (**Figures 3**, **4**, **Tables 2**, **4**).

**Table 4:** Anemia and malignancy– related age-adjusted mortality rates per 100,000, Stratified by race in older adults in the United States, 1999 to 2020.

Year	Black or African American	NH American Indian or Alaska native	Hispanic or latino	NH Asian or Pacific Islander	NH white
1999	54.49 (51.69–57.29)	21.86 (14.01–32.53)	26.66 (23.95–29.36)	26.79 (22.70–30.88)	29.72 (29.09–30.34)
2000	54.71 (51.91–57.50)	22.55 (15.10–32.39)	24.50 (21.96–27.04)	26.78 (22.91–30.66)	28.94 (28.32–29.55)
2001	51.92 (49.21–54.63)	28.18 (19.51–39.37)	24.98 (22.50–27.46)	24.71 (21.16–28.26)	28.79 (28.18–29.39)
2002	52.25 (49.53–54.96)	32.08 (22.81–43.85)	26.13 (23.65–28.61)	24.23 (20.78–27.67)	28.82 (28.21–29.42)
2003	50.61 (47.96–53.26)	35.27 (25.73–47.20)	23.88 (21.59–26.18)	23.88 (20.63–27.12)	28.46 (27.86–29.06)
2004	48.57 (45.99–51.15)	26.84 (18.59–37.51)	23.61 (21.38–25.85)	26.83 (23.47–30.19)	28.24 (27.64–28.83)
2005	44.70 (42.26–47.14)	24.33 (16.95–33.84)	25.60 (23.35–27.85)	23.26 (20.23–26.30)	27.74 (27.16–28.33)
2006	45.34 (42.89–47.78)	29.03 (20.92–39.24)	24.80 (22.64–26.97)	22.87 (19.95–25.79)	28.19 (27.61–28.78)
2007	46.00 (43.57–48.43)	29.29 (21.20–39.46)	25.31 (23.18–27.43)	23.57 (20.71–26.44)	27.13 (26.56–27.70)
2008	45.28 (42.89–47.66)	27.85 (20.23–37.38)	22.76 (20.80–24.72)	24.90 (22.03–27.77)	27.89 (27.31–28.46)
2009	42.13 (39.85–44.40)	25.63 (18.39–34.77)	23.26 (21.33–25.19)	24.70 (21.93–27.47)	26.80 (26.24–27.36)
2010	43.13 (40.86–45.40)	39.53 (30.51–50.38)	24.27 (22.34–26.20)	26.19 (23.41–28.98)	28.29 (27.71–28.86)
2011	41.00 (38.83–43.18)	31.71 (23.89–41.27)	23.68 (21.85–25.51)	23.48 (20.96–25.99)	27.08 (26.52–27.63)
2012	40.08 (37.97–42.19)	32.81 (25.04–42.23)	26.50 (24.62–28.37)	23.96 (21.48–26.43)	27.26 (26.71–27.82)
2013	38.10 (36.08–40.12)	29.95 (22.85–38.55)	25.73 (23.93–27.53)	20.74 (18.54–22.95)	27.19 (26.64–27.73)
2014	38.82 (36.83–40.82)	28.22 (21.48–36.40)	23.66 (21.99–25.33)	22.52 (20.31–24.73)	26.48 (25.95–27.02)
2015	35.57 (33.70–37.45)	20.67 (15.19–27.49)	23.31 (21.70–24.92)	20.40 (18.38–22.42)	25.90 (25.37–26.42)
2016	34.47 (32.66–36.28)	21.00 (15.59–27.69)	23.88 (22.29–25.47)	16.91 (15.13–18.70)	26.22 (25.70–26.75)
2017	36.79 (34.96–38.63)	27.14 (21.16–34.29)	23.83 (22.29–25.37)	20.84 (18.91–22.76)	26.98 (26.45–27.50)
2018	35.11 (33.36–36.86)	23.01 (17.60–29.56)	23.01 (21.53–24.48)	20.07 (18.23–21.91)	27.90 (27.37–28.42)
2019	34.25 (32.56–35.94)	27.42 (21.81–34.03)	23.88 (22.40–25.35)	20.57 (18.76–22.38)	28.28 (27.75–28.80)
2020	41.13 (39.30–42.96)	28.31 (22.73–34.84)	24.84 (23.37–26.31)	19.68 (17.97–21.39)	31.19 (30.65–31.74)
total	42.39 (41.93–42.86)	27.53 (25.91–29.15)	24.25 (23.85–24.64)	22.16 (21.64–22.68)	27.88 (27.76–28.00)

### 3.4 Anemia and malignancy-related AAMR Stratified by geographical regions

**Figure 5:**
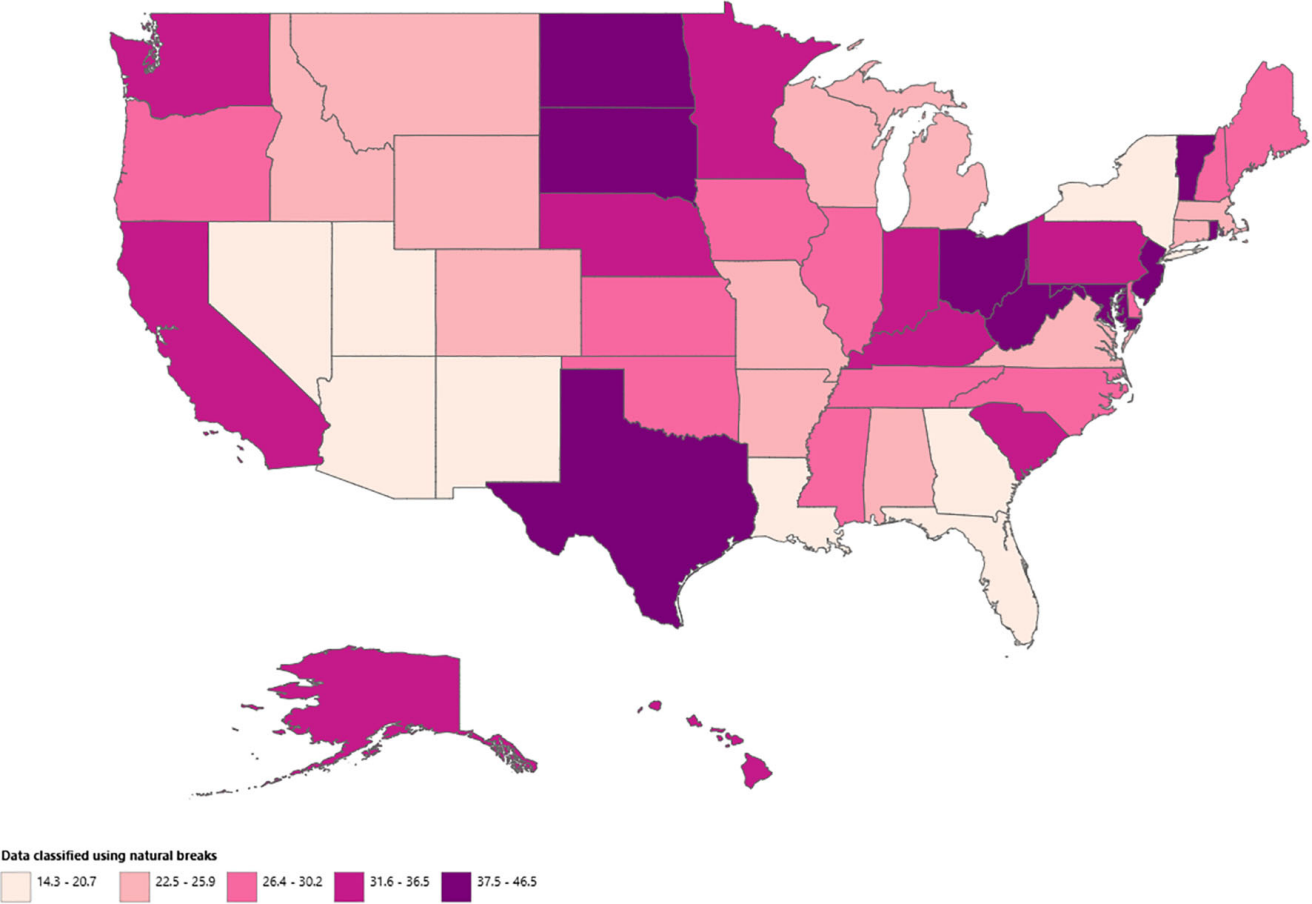
States stratified anemia and malignancy related age-adjusted mortality rates per 100,000 among older adults aged ≥65 years in the United States, 1999–2020.

Geographic disparities in AAMR were substantial, with state-level rates ranging from 14.33 (95% CI: 13.43 to 15.24) in Nevada to 46.49 (95% CI: 43.73 to 49.25) in North Dakota. States within the top 90th percentile for AAMRs included Maryland, North Dakota, Rhode Island, West Virginia, and the District of Columbia, each recording nearly 1.5 times the rates of those in the bottom 10th percentile, which included Louisiana, Arizona, Georgia, Nevada, and Utah. (**Supplementary Table 2**) These regional trends are visually represented on the WONDER Map using quantile classification (**Figure 5**).

**Figure 6:**
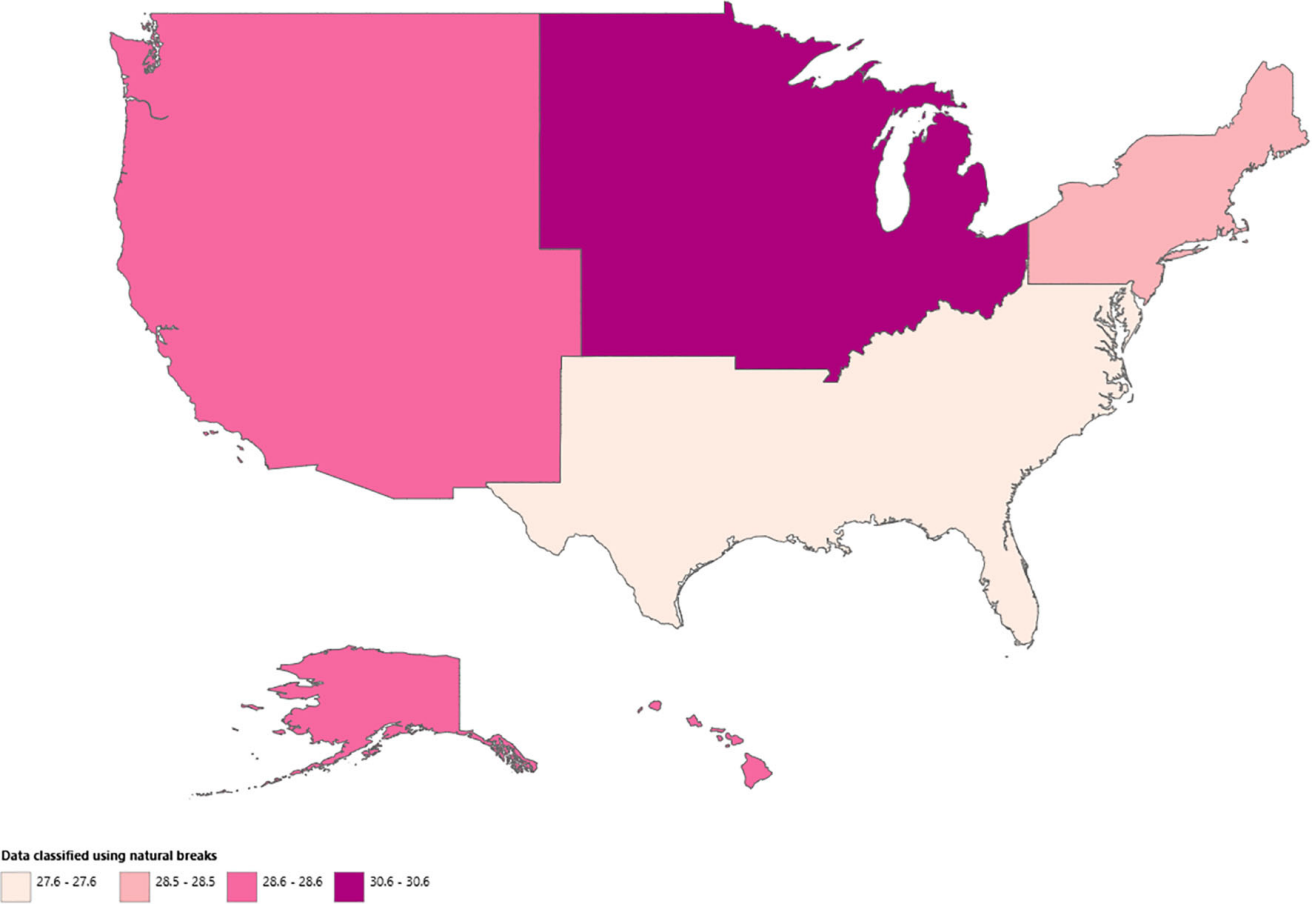
Census region stratified anemia and malignancy related age-adjusted mortality rates per 100,000 among older adults aged ≥65 years in the United States, 1999–2020.

Regionally, the highest mortality rates over the study period were observed in the Midwest (AAMR: 30.64; 95% CI: 30.40 to 30.88), followed by the West (AAMR: 28.62; 95% CI: 28.38 to 28.85), Northeast (AAMR: 28.46; 95% CI: 28.22 to 28.71), and South (AAMR: 27.64; 95% CI: 27.46 to 27.82). (**Figure 6**, **Table 2**, **Supplementary Table 3**). During the study period, the Northeast was the only region to exhibit a consistent downward trend in AAMR (AAPC: -0.95; 95% CI: -1.22 to -0.68). In contrast, both the Midwest and South experienced a steady decline in AAMR from 1999 to 2016 (APC: Midwest -1.49; 95% CI: -1.69 to -1.28; South -0.66; 95% CI: -0.89 to -0.43), followed by a significant increase through 2020 (APC: Midwest 4.18; 95% CI: 2.35 to 6.04; South 4.70; 95% CI: 2.88 to 6.55). In the West, AAMR declined from 1999 to 2018 (APC: -0.48; 95% CI: -0.80 to -0.16), after which it remained stable through 2020 (APC: 5.18; 95% CI: -3.94 to 15.17). Overall, AAPC was -0.43 (95% CI: -0.78 to -0.08) for the Midwest, 0.34 (95% CI: -0.01 to 0.70; p>0.05) for the South, and 0.05 (95% CI: -0.80 to 0.90; p>0.05) for the West.

**Figure 7:**
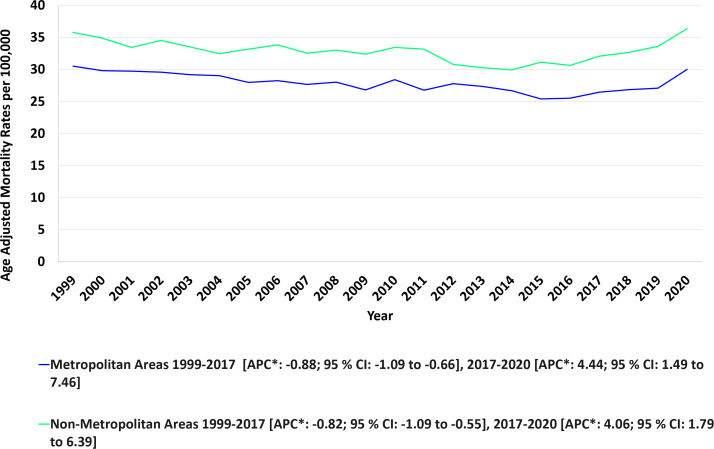
Urbanization stratified anemia and malignancy related age-adjusted mortality rates per 100,000 among older adults aged ≥65 years in the United States, 1999–2020.* Indicates that the annual percentchange (APC) is significantly different from zero at α = 0.05. AAMR, age-adjusted mortality rate; APC, annual percent change; CI, confidence interval.

Non-metropolitan areas consistently experienced higher AAMRs than metropolitan areas (Non-metropolitan: 32.89; 95% CI: 32.61 to 33.16; Metropolitan: 27.81; 95% CI: 27.69 to 27.93). Between 1999 and 2017, metropolitan AAMRs declined steadily (APC: -0.88; 95% CI: -1.09 to -0.66), followed by a notable rise from 2017 to 2020 (APC: 4.44; 95% CI: 1.49 to 7.46). For non-metropolitan areas, AAMRs also experienced a downward trend from 1999 to 2016 (APC: -0.82; 95% CI: -1.09 to -0.55), before increasing through 2020 (APC: 4.06; 95% CI: 1.79 to 6.39). (**Figure 7**, **Table 5**). During the study period, AAPC for metropolitan areas was -0.13 (95% CI: -0.55 to 0.28; p>0.05) and 0.09 (95% CI: -0.35 to 0.53; p>0.05) for non-metropolitan areas.

**Table 5:** Anemia and malignancy– related age-adjusted mortality rates per 100,000, stratified by urban-rural classification in older adults in the United States, 1999 to 2020.

Year	Metropolitan areas	Non metropolitan areas
1999	30.51 (29.86–31.16)	35.75 (34.32–37.19)
2000	29.79 (29.15–30.43)	34.85 (33.44–36.26)
2001	29.73 (29.10–30.36)	33.43 (32.05–34.80)
2002	29.56 (28.93–30.19)	34.53 (33.13–35.93)
2003	29.18 (28.56–29.80)	33.49 (32.12–34.87)
2004	29.00 (28.39–29.62)	32.46 (31.11–33.81)
2005	27.97 (27.38–28.57)	33.13 (31.78–34.48)
2006	28.25 (27.65–28.84)	33.82 (32.46–35.18)
2007	27.63 (27.05–28.21)	32.53 (31.21–33.86)
2008	28.00 (27.42–28.58)	32.98 (31.66–34.31)
2009	26.77 (26.20–27.33)	32.35 (31.04–33.65)
2010	28.38 (27.80–28.95)	33.42 (32.10–34.75)
2011	26.76 (26.21–27.31)	33.15 (31.84–34.45)
2012	27.77 (27.21–28.32)	30.78 (29.54–32.03)
2013	27.32 (26.78–27.86)	30.25 (29.03–31.48)
2014	26.68 (26.16–27.21)	29.92 (28.71–31.13)
2015	25.40 (24.89–25.91)	31.12 (29.90–32.34)
2016	25.51 (25.01–26.02)	30.60 (29.40–31.80)
2017	26.42 (25.92–26.93)	32.05 (30.84–33.27)
2018	26.84 (26.34–27.34)	32.62 (31.41–33.83)
2019	27.08 (26.58–27.57)	33.56 (32.35–34.78)
2020	29.99 (29.48–30.50)	36.36 (35.11–37.62)

### 3.5 Subgroup analysis by cancer type

From 1999 to 2020, various malignancies exhibited a significant downward trend, albeit with some variability among them. CRC demonstrated the steepest decline (AAPC: -2.03; 95%CI: -2.45 to −1.60, p-value <0.05); followed by gastric cancer (AAPC: -1.96; 95% CI: -2.66 to -1.26) and prostate cancer (AAPC: 1.40; 95%CI: -2.02 to -0.77). BC, lung, gynecological, and hematological cancers showed a non-significant change over the study period. Lung cancer: [AAPC: 0.96: 95% CI: -0.20 to 2.13 (p-value=0.1)]; BC: [AAPC: 0.12; 95% CI: -0.73 to 0.99 (p value= 0.7)]; Gynecological cancer: [AAPC: 0.55; 95% CI: -0.20 to 1.30 (p value= 0.15)]; Hematological cancer: [AAPC: 0.02; 95% CI: -0.59 to 0.64 (p value= 0.943)].

**Figure 8:**
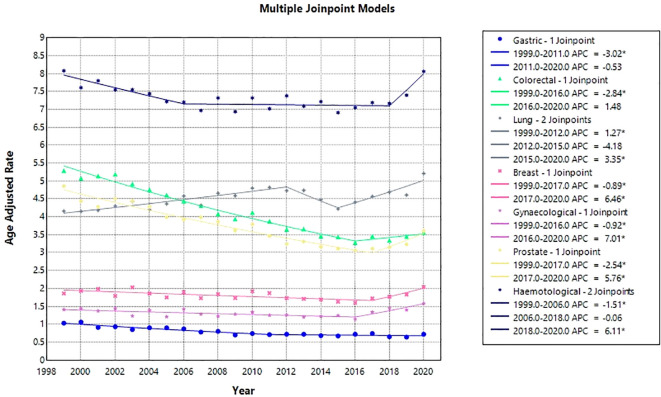
Joinpoint model of anemia and malignancy-related AAMR per 100,000; stratified by cancer type, 1999-2020 (*indicates the APC is statistically significant).

**Figure 9:**
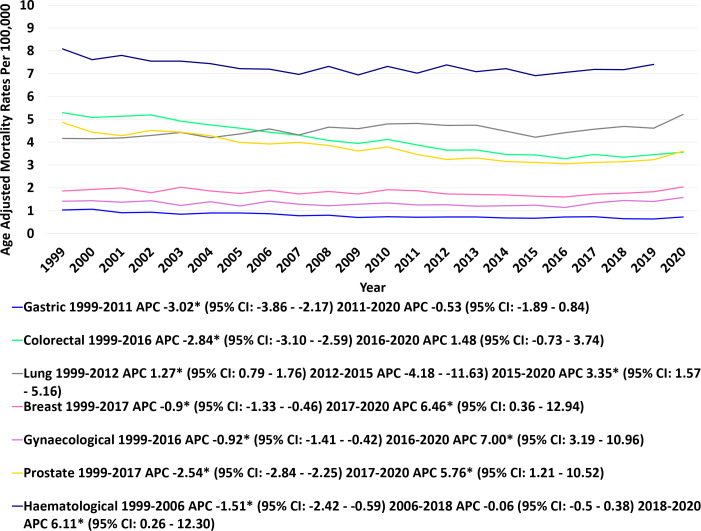
Anemia and malignancy related age-adjusted mortality rates per 100,000 among older adults aged ≥65 years in the United States, stratified by cancer type, 1999–2020.* Indicates that the annual percent change (APC) is significantly different from zero at α = 0.05. AAMR, age-adjusted mortality rate; APC, annual percent change; CI, confidence interval.

CRC demonstrated a significant decline in AAMR from 1999 to 2016 (APC: –2.84; 95% CI: –3.09 to –2.58), followed by a plateau through 2020 (APC: 1.48; 95% CI: –0.73 to 3.74; P = 0.176). Gynecologic cancers also showed a steady decline from 1999 to 2016 (APC: –0.92; 95% CI: –1.41 to –0.42), but this was followed by a marked increase from 2016 to 2020 (APC: 7.01; 95% CI: 3.19 to 10.96). Prostate cancer mortality declined from 1999 to 2017 (APC: –2.54; 95% CI: –2.84 to –2.25), after which a significant rise was observed from 2017 to 2020 (APC: 5.76; 95% CI: 1.21 to 10.52). Similarly, BC mortality decreased steadily from 1999 to 2017 (APC: –0.89; 95% CI: –1.33 to –0.46), but reversed to a significant increase from 2017 to 2020 (APC: 6.46; 95% CI: 0.36 to 12.94). Gastric cancer showed a significant decline from 1999 to 2011 (APC: –3.02; 95% CI: –3.86 to –2.17), followed by stability until 2020 (APC: –0.53; 95% CI: –1.89 to 0.84; P = 0.42). Lung cancer exhibited a distinct pattern, with an initial steady increase from 1999 to 2012 (APC: 1.27; 95% CI: 0.78 to 1.76), a period of stability from 2012 to 2015 (APC: –4.18; 95% CI: –11.62 to 3.90; P = 0.28), and a subsequent sharp rise from 2015 to 2020 (APC: 3.35; 95% CI: 1.57 to 5.16). Hematological cancers declined steadily from 1999 to 2006 (APC: –1.51; 95% CI: –2.42 to –0.60), remained stable from 2006 to 2018 (APC: –0.06; 95% CI: –0.50 to 0.38; P = 0.77), and then reversed to a significant increase in mortality from 2018 to 2020 (APC: 6.11; 95% CI: 0.26 to 12.30) (**Figures 8**, **9**).

## 4 Discussion

**Figure 10:**
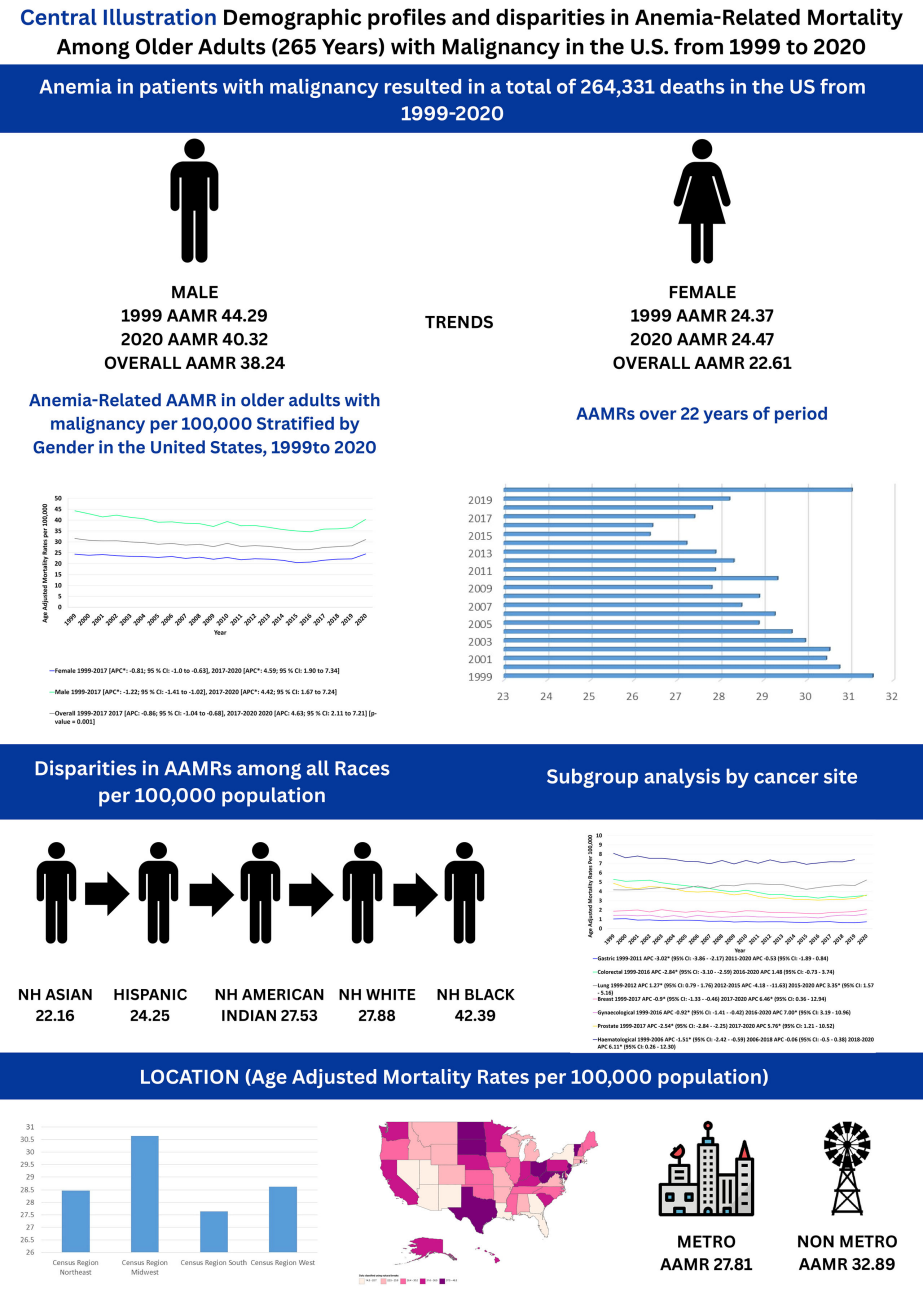
Central illustration summarizing key trends in anemia and malignancy-related age-adjusted mortality rates per 100,000 among older adults aged ≥65 years in the United States, 1999–2020. AAMR, age-adjusted mortality rate; NH, non-Hispanic.

In this 22-year CDC mortality analysis, several key patterns emerged. AAMRs declined from 1999 to 2017, but rose sharply thereafter through 2020 with consistently higher rates among males than females. Racial disparities were evident, as NH Black or African American individuals had the highest AAMRs, while Asian or Pacific Islanders had the lowest. Geographic variation was also marked: states in the top 90th percentile (e.g., Maryland, North Dakota, Rhode Island) recorded nearly 1.5-fold higher AAMRs than those in the lowest decile (e.g., Louisiana, Arizona, Georgia, Nevada, Utah). The Midwest and non-metropolitan regions bore the greatest burden. By cancer type, CRC and gastric cancers showed the most pronounced and sustained mortality declines, whereas BC, gynecologic, prostate, lung, and hematologic cancers demonstrated initial decreases followed by concerning increases in recent years (**Figure 10**).

Mortality associated with concurrent anemia and malignancy is reported to be highest among individuals over 75 years of age, partly due to reduced physiological tolerance to anemia (31). Moreover, increased eryptosis, programmed erythrocyte death has been observed in the elderly, leading to greater red blood cell loss and worsening anemia (32). The initial decline in anemia and malignancy-related mortality from 1999 to 2017 may likely reflect advances in early cancer detection, the introduction of more effective systemic therapies, and improvements in supportive care, including erythropoiesis-stimulating agents (ESAs), iron supplementation, and optimized transfusion practices guided by ASCO/ASH recommendations introduced in 2010 (33). These interventions might have potentially helped reduce anemia severity and improve patient outcomes.

The post-2017 resurgence in mortality may likely reflect a convergence of therapeutic shifts, administrative changes, rising frailty and multimorbidity, and the acute shock of the COVID-19 pandemic. The U.S. transition from ICD-9-CM to ICD-10-CM in 2015 increased diagnostic specificity, and as clinicians adapted, more granular electronic health record (EHR) problem lists led to more frequent documentation of comorbidities such as anemia, subsequently appearing more often on death certificates and contributing to an administrative rise in mortality (34, 35). This may also have been compounded by the long-term “lag effect” of restrictive ESA policies; following the 2011 FDA Risk Evaluation and Mitigation Strategy (REMS), ESA use declined sharply (33, 36), leaving many older, frail adults with undertreated chronic anemia and potentially poorer long-term outcomes. Concurrently, the therapeutic landscape shifted with the widespread adoption of immune checkpoint inhibitors (ICIs), which, despite improving survival, introduced immune-related hematologic toxicities, including hemolytic anemia and cytopenias, potentially increasing the complexity of clinical management in older adults (37). The rising mortality trend may also reflect the growing multimorbidity burden among aging cancer patients. National data show that the prevalence of multiple chronic conditions among older U.S. adults increased substantially, independent of cancer status (38). As oncology care increasingly involves adults over 80 years with significant cardiovascular, renal, and metabolic comorbidities, anemia becomes closely intertwined with broader frailty trajectories (39). Even mild anemia has been associated with functional decline and cardiac stress, features termed “anemia-associated frailty” (31, 39). Thus, the late-period mortality rise may align with an older, more comorbid population living longer with cancer but accumulating greater physiological vulnerability (40).

The sharp 2019–2020 mortality escalation likely reflects interacting biological and administrative factors. While this dataset cannot confirm specific biomarkers, literature suggests that biologically, older cancer patients may have faced a “double hit”: beyond the direct mortality of viral infection, the COVID-19 “cytokine storm” elevates interleukin-6 (IL-6) and hepcidin, sequestering iron and blocking erythropoiesis. This mechanism, described in prior studies, potentially exacerbates pre-existing anemia in cancer patients and might precipitate lethal hematologic decompensation in already compromised marrow (41–43). This synergy may have been further aggravated by systemic delays in blood transfusions and supportive care (44). Administratively, the rise might have been amplified by 2020 National Center for Health Statistics (NCHS) guidance, explicitly encouraging certifiers to detail the “chain of events” and include all “significant conditions contributing to death” in Part II of the death certificate to fully capture the pandemic’s risk profile (45). This increased documentation of “background” comorbidities like anemia suggests the 2020 peak may represent a composite of true excess mortality and a surveillance artifact.

Our study demonstrated that men consistently exhibited higher AAMRs than women, aligning with reports of higher anemia prevalence and cancer incidence among male oncology cohorts, which may partly explain their increased mortality (46). This disparity may also reflect men’s lower physiological tolerance to anemia and greater susceptibility to hypoxia at comparable hemoglobin levels (47). Furthermore, males generally experience more aggressive cancer progression and poorer survival outcomes, influenced by a combination of biological, behavioral, and treatment-related factors (48).In contrast, females appear to benefit from several protective mechanisms, including estrogen-mediated immune enhancement, more effective tumor surveillance, genetic advantages from dual X chromosomes, and greater engagement in health care and treatment adherence (49–51). For instance, “escape from X-inactivation tumor suppressor” (EXITS) genes—such as *MAGEC3, KDM6A, KDM5C, DDX3X, CNKSR2*, and *ATRX*- provide females with biallelic tumor-suppressive protection, whereas males carry only a single copy. Many of these genes also regulate *p53* activity, a mechanism cited in literature that may contribute to the higher prevalence of *p53* mutations seen in male cancers (52).

Higher mortality among NH Black populations likely reflects structural racism and social determinants of health (SDOH) interacting with biological risks. Beyond treatment differences (53, 54), cumulative social stress (“weathering”) may accelerate biological aging, increasing vulnerability to cancer and anemia (55). Structural barriers, including lower screening, delayed hematologic referrals, and reduced supportive care (56, 57), as well as residence in environmental justice zones with high pollutant exposure (58) cultural mistrust and underrepresentation in research (59) further exacerbate disparities. In contrast, lower mortality in Asian American populations may relate to distinct cancer epidemiology and genetic profiles, such as higher prevalence of EGFR mutations responsive to targeted therapies (60), alongside socioeconomic factors.

Pronounced regional and urban–rural disparities were evident, with nonmetropolitan populations consistently experiencing higher mortality. These differences likely reflect structural workforce shortages limiting access to specialized oncology care, transportation barriers, compounded by travel distance and limited public transit infrastructure that reduce adherence to complex treatment regimens and supportive services (61, 62). National data further indicate higher mortality across multiple conditions in rural areas, reflecting broader disparities in healthcare access, screening, and treatment, including higher mastectomy rates and lower radiation use (63, 64). Regionally, the Northeast showed sustained mortality declines that may likely be due to better healthcare access, early detection, and improved anemia management (65). In contrast, the Midwest and South experienced post-2016 increases, which may have been influenced by socioeconomic inequities, healthcare disruptions, delayed diagnoses, higher comorbidity burdens, and limited oncology resources (66). The West exhibited modest declines with a late plateau, occurring alongside regional variations in cancer subtypes and supportive care practices. Overall, these findings highlight persistent geographic inequities and underscore the need for equity-focused strategies to improve early detection, expand access to care, and optimize outcomes nationwide.

In our subgroup analysis, we observed heterogeneous cancer-specific trends. CRC, gastric, and prostate cancers demonstrated significant overall declines in mortality, with CRC showing the steepest reduction until 2016, followed by gastric and prostate cancer. Notably, lung cancer showed the most dynamic trajectory, alternating between periods of rise, decline, and resurgence. These findings highlight the complexity of concurrent anemia and cancer related mortality and underscore the need to contextualize each trend within the framework of evolving screening practices, therapeutic advances, and comorbid burden. The decline in CRC, gastric, and prostate cancer mortality temporally coincides with public health and clinical interventions. CRC screening, formally recommended by the American Cancer Society (ACS) from 2001 onward and supported by Medicare coverage, led to widespread adoption of colonoscopy, fecal immunochemical testing (FIT), and sigmoidoscopy at defined intervals for individuals aged 50 years and older. Population-level evidence shows screening reduces CRC mortality by over 50% through early detection and polyp removal (67–69). Our findings suggest that the mortality decline observed in the 2010s may have been associated with these structured screening practices. Mortality plateauing after 2016 likely reflects screening saturation and rising incidence in younger adults (70).

Gastric cancer mortality has declined due to multiple public health and clinical advances, including reduced Helicobacter pylori prevalence, improved food preservation, lower smoking and salt intake, and enhanced sanitation, collectively decreasing both incidence and death rates (71, 72). Prostate cancer mortality also fell between 1999 and 2017, that may have largely been driven by prostate-specific antigen (PSA) screening introduced in the late 1980s, which facilitated earlier detection and stage migration (67, 73). However, after 2017, AAMR rose, potentially reflecting the 2012 United States Preventive Services Task Force (USPSTF) Grade D recommendation against routine PSA screening, which may have reduced early diagnosis and increased metastatic presentations at detection (74). Similarly, BC mortality declined from 1999 to 2017, potentially aided by widespread mammography screening and advances in systemic therapies, with the reporting a 44% reduction in deaths since 1989 (75). Yet, in this cohort with concurrent anemia, mortality increased after 2017. A 2025 study indicated that 85% of BC diagnosed in 2017 were stage I or II, typically low-risk yet these now account for a substantial proportion of deaths, suggesting that factors beyond tumor stage, including treatment delays, comorbidities, or barriers to optimal care, may contribute (76). Gynecological cancers generally declined from 1999 to 2016, which may have driven by effective cervical cancer screening through Pap smears and HPV testing (68), as well as HPV vaccination beginning in 2008 (77). Cancers lacking routine screening, such as ovarian and some other gynecological cancers, have experienced rising mortality since 2017. For example, uterine cancer deaths increased by 1.5% per year from 2013 to 2022, with similar upward trends observed in ovarian cancer.

Hematological cancers exhibited a biphasic mortality pattern: a steep decline from 1999 to 2006, a plateau from 2006 to 2018, and an increase after 2018. The early decline maybe temporally associated with the introduction of rituximab in 1997, which revolutionized B-cell malignancy treatment (78). The subsequent plateau likely reflects limits of initial therapeutic advances and the emergence of therapy-resistant disease subtypes. The recent rise in mortality may be partly attributable to the COVID-19 pandemic, as patients with hematologic malignancies are particularly vulnerable to severe SARS-CoV-2 infection (42). Lung cancer exhibited a dynamic, biphasic mortality pattern in anemic patients. Mortality rose from 1999 to 2012, largely reflecting the cumulative effects of historical smoking behaviors, as tobacco use remains the leading risk factor for lung cancer (79, 80). From 2012, mortality stabilized, coinciding with the 2013 USPSTF recommendation for annual low-dose CT screening in high-risk adults, although slow uptake, limited measurable impact (80). A subsequent sharp rise from 2015 to 2020 coincided with the COVID-19 pandemic, which disproportionately increased mortality among lung cancer patients (43). This increase in mortality is also attributable to smoking, increases with advancing age and is influenced by birth cohort effects, indicating the long-term impact of smoking behaviors (81).

### 4.1 Limitations

This study has several limitations. Reliance on death certificates and ICD-10 coding may have led to potential misclassification or underreporting of anemia and malignancy as causes of death (82). Anemia is often viewed as a clinical sign rather than a distinct cause of death; therefore, it is likely only recorded when severe or directly contributing to the terminal event, suggesting our rates are conservative estimates. Variation in coding practices across states and over time, such as differences between coroner and medical examiner systems may influence observed geographic trends (83). As an ecological study relying on aggregate data, we were unable to perform multivariate adjustments for key patient-level confounders. The absence of data on socioeconomic status, **s**pecific insurance type (e.g., Medicare Advantage vs. Fee-for-Service), healthcare access, facility-level oncologic capacity, and lifestyle introduces potential residual confounding. Consequently, the disparities reported herein represent unadjusted associations and may be influenced by these unmeasured effect modifiers. The CDC WONDER database also lacks detailed clinical information, such as hemoglobin levels, cancer stage, treatment regimens, and specific comorbidity indices, which precludes causal inference regarding the impact of specific interventions.Temporal changes in diagnostic criteria, coding practices, and reporting accuracy may also have influenced observed trends. While we noted that estimates for smaller groups like NH American Indian or Alaska Natives may be unstable due to sample sizes, we must also acknowledge racial misclassification as a distinct source of bias. Previous studies indicate that AI/AN and Asian/Pacific Islander individuals are sometimes misclassified as White or “Other” on death records, which may lead to an underestimation of mortality rates in these specific populations distinct from sample size issues (84). Reliance on death certificate data captures the co-occurrence of anemia and malignancy codes but cannot definitively establish clinical etiology. Consequently, this study identifies ‘anemia in the setting of malignancy’ and may include cases of incidental or multifactorial anemia not strictly caused by the tumor or its treatment.while we discuss potential biological mechanisms such as inflammatory cytokine activity, this epidemiological dataset does not contain biomarker data; therefore, these associations remain hypothesis-generating. Nevertheless, despite these limitations, the use of a large, nationally representative dataset spanning over two decades provides valuable insights into long-term population trends, enabling the identification of meaningful disparities in anemia and malignancy related mortality that warrant targeted public health and clinical intervention.

## 5 Conclusion

Between 1999 and 2020, anemia and malignancy-related mortality among U.S. adults aged ≥65 exhibited an initial decline followed by a significant resurgence in recent years. These trends were associated with persistent demographic and geographic disparities, with the highest rates observed among males, NH black individuals, and residents of nonmetropolitan areas. While CRC and gastric cancers showed sustained declines, the recent reversals in BC, prostate, and lung cancer mortality likely reflect a complex interplay of administrative coding shifts, an aging multimorbid population, and therapeutic changes. These findings underscore the importance of targeted public health strategies to address the structural and regional inequities characterizing the burden of anemia in older cancer patients.

## Data Availability

The original contributions presented in the study are included in the article/**Supplementary Material**. Further inquiries can be directed to the corresponding author.
