# Supplementary material for: Regional, demographic and temporal trends in anemia and malignant cancer-related mortality in U.S. older adults: a nationwide CDC WONDER analysis (1999–2020)
Source: Front Oncol. 2026 Jan 21;16:1722891. doi: 10.3389/fonc.2026.1722891 (PMC12867818; doi:10.3389/fonc.2026.1722891)
Supplement: Supplementary file 1 [file Table1.docx]

**Supplementary material**

**Supplemental Table 1:** Total number of anemia and malignancy-related deaths among older adults (≥65 years) in the United States from 1999 to 2020, stratified by Place of Death (including medical facilities, decedent’s home, hospice facilities, and nursing homes).

| **Year** | **Medical facility** | **Nursing home or long term care facility** | **Hospice** | **Home** | **Place of death unkown** | **Other** |
| --- | --- | --- | --- | --- | --- | --- |
| 1999 | 4643 | 3288 | missing | 2565 | missing | 333 |
| 2000 | 4560 | 3273 | missing | 2486 | missing | 353 |
| 2001 | 4444 | 3297 | missing | 2539 | missing | 416 |
| 2002 | 4473 | 3346 | missing | 2583 | missing | 426 |
| 2003 | 4401 | 3369 | 31 | 2609 | 30 | 399 |
| 2004 | 4416 | 3230 | 55 | 2639 | 28 | 468 |
| 2005 | 4203 | 3271 | 148 | 2636 | 23 | 465 |
| 2006 | 4365 | 3225 | 195 | 2809 | 39 | 439 |
| 2007 | 4184 | 3266 | 290 | 2749 | 20 | 467 |
| 2008 | 4418 | 3196 | 339 | 2844 | 118 | 441 |
| 2009 | 4225 | 3000 | 378 | 2872 | 197 | 445 |
| 2010 | 4351 | 3239 | 453 | 3279 | missing | 558 |
| 2011 | 4477 | 3010 | 507 | 3142 | missing | 522 |
| 2012 | 4482 | 3038 | 659 | 3418 | missing | 541 |
| 2013 | 4430 | 2959 | 730 | 3611 | missing | 552 |
| 2014 | 4534 | 3049 | 737 | 3602 | 10 | 440 |
| 2015 | 4541 | 2862 | 834 | 3685 | missing | 408 |
| 2016 | 4729 | 2671 | 916 | 3924 | missing | 402 |
| 2017 | 4873 | 2823 | 1165 | 4150 | missing | 446 |
| 2018 | 5117 | 2831 | 1258 | 4359 | missing | 532 |
| 2019 | 5497 | 2712 | 1379 | 4575 | missing | 528 |
| 2020 | 5413 | 2420 | 1465 | 6626 | missing | 664 |
| total | 10076 | 67375 | 11539 | 73702 | 517 | 10245 |

**Supplemental Table 2;** Age-adjusted mortality rates (AAMRs) per 100,000 population for concurrent anemia and malignancy among adults aged ≥65 years in the United States from 1999 to 2020, stratified by State, with corresponding 95% confidence intervals to support geographic distribution maps.

| **States** | **AAMR (95% CI)** |
| --- | --- |
| Nevada | 14.33 (13.43–15.24) |
| Arizona | 15.55 (15.00–16.10) |
| Louisiana | 16.18 (15.47–16.88) |
| Utah | 16.26 (15.20–17.33) |
| Georgia | 19.38 (18.80–19.96) |
| Florida | 20.40 (20.08–20.72) |
| New Mexico | 20.56 (19.40–21.72) |
| New York | 20.71 (20.35–21.08) |
| Montana | 22.53 (20.91–24.15) |
| Massachusetts | 22.58 (21.95–23.22) |
| Idaho | 22.60 (21.17–24.02) |
| Virginia | 23.01 (22.37–23.65) |
| Alabama | 23.35 (22.56–24.14) |
| Colorado | 23.63 (22.77–24.50) |
| Wisconsin | 24.34 (23.62–25.06) |
| Missouri | 25.08 (24.37–25.79) |
| Michigan | 25.18 (24.62–25.74) |
| Arkansas | 25.19 (24.17–26.21) |
| Wyoming | 25.70 (23.16–28.24) |
| Connecticut | 25.93 (25.02–26.84) |
| Maine | 26.45 (24.99–27.90) |
| Delaware | 26.54 (24.64–28.45) |
| Kansas | 26.81 (25.74–27.89) |
| Mississippi | 26.99 (25.88–28.10) |
| Oregon | 27.22 (26.30–28.15) |
| Illinois | 27.84 (27.30–28.37) |
| Oklahoma | 28.24 (27.26–29.23) |
| North Carolina | 29.20 (28.55–29.84) |
| Tennessee | 29.95 (29.16–30.74) |
| Iowa | 30.16 (29.13–31.20) |
| New Hampshire | 30.18 (28.49–31.87) |
| Hawaii | 31.55 (29.91–33.19) |
| Kentucky | 32.07 (31.08–33.06) |
| Washington | 32.65 (31.83–33.47) |
| South Carolina | 33.02 (32.05–33.99) |
| Indiana | 33.68 (32.85–34.50) |
| California | 33.75 (33.38–34.11) |
| Alaska | 34.74 (31.11–38.37) |
| Pennsylvania | 35.23 (34.69–35.77) |
| Nebraska | 36.48 (34.93–38.03) |
| Minnesota | 36.52 (35.58–37.45) |
| Texas | 37.52 (37.02–38.02) |
| New Jersey | 37.83 (37.11–38.56) |
| Vermont | 38.08 (35.41–40.75) |
| Ohio | 38.29 (37.66–38.92) |
| South Dakota | 38.72 (36.42–41.03) |
| Rhode Island | 39.03 (37.02–41.04) |
| Maryland | 39.18 (38.21–40.15) |
| West Virginia | 39.42 (37.90–40.94) |
| District of Columbia | 39.76 (36.66–42.85) |
| North Dakota | 46.49 (43.73–49.25) |

**Supplemental Table 3;** Age-adjusted mortality rates (AAMRs) per 100,000 population for concurrent anemia and malignancy among adults aged ≥65 years in the United States from 1999 to 2020, stratified by Census region, with corresponding 95% confidence intervals to illustrate regional disparities.

| **Census region** | **Year** | **AAMR (95% CI)** |
| --- | --- | --- |
| Northeast | 1999 | 32.63 (31.33–33.93) |
| Northeast | 2000 | 31.69 (30.41–32.97) |
| Northeast | 2001 | 31.13 (29.87–32.39) |
| Northeast | 2002 | 31.65 (30.38–32.91) |
| Northeast | 2003 | 29.68 (28.46–30.90) |
| Northeast | 2004 | 30.33 (29.10–31.56) |
| Northeast | 2005 | 29.40 (28.19–30.61) |
| Northeast | 2006 | 28.33 (27.15–29.51) |
| Northeast | 2007 | 28.05 (26.88–29.22) |
| Northeast | 2008 | 29.24 (28.05–30.42) |
| Northeast | 2009 | 26.78 (25.65–27.91) |
| Northeast | 2010 | 29.86 (28.67–31.05) |
| Northeast | 2011 | 27.90 (26.75–29.04) |
| Northeast | 2012 | 29.35 (28.19–30.52) |
| Northeast | 2013 | 28.12 (27.00–29.25) |
| Northeast | 2014 | 27.53 (26.43–28.64) |
| Northeast | 2015 | 26.45 (25.37–27.53) |
| Northeast | 2016 | 26.12 (25.06–27.18) |
| Northeast | 2017 | 25.78 (24.73–26.82) |
| Northeast | 2018 | 26.40 (25.35–27.44) |
| Northeast | 2019 | 24.73 (23.73–25.73) |
| Northeast | 2020 | 28.32 (27.26–29.38) |
| Northeast | total | 28.46 (28.22–28.71) |
| Midwest | 1999 | 34.60 (33.34–35.87) |
| Midwest | 2000 | 33.63 (32.39–34.87) |
| Midwest | 2001 | 34.33 (33.08–35.57) |
| Midwest | 2002 | 33.18 (31.96–34.40) |
| Midwest | 2003 | 34.47 (33.23–35.71) |
| Midwest | 2004 | 32.38 (31.18–33.58) |
| Midwest | 2005 | 32.19 (31.00–33.37) |
| Midwest | 2006 | 31.65 (30.48–32.83) |
| Midwest | 2007 | 31.02 (29.87–32.17) |
| Midwest | 2008 | 31.55 (30.40–32.71) |
| Midwest | 2009 | 29.73 (28.61–30.84) |
| Midwest | 2010 | 29.89 (28.77–31.00) |
| Midwest | 2011 | 28.89 (27.80–29.98) |
| Midwest | 2012 | 28.67 (27.60–29.75) |
| Midwest | 2013 | 28.28 (27.22–29.33) |
| Midwest | 2014 | 27.08 (26.05–28.10) |
| Midwest | 2015 | 27.90 (26.87–28.94) |
| Midwest | 2016 | 27.71 (26.69–28.73) |
| Midwest | 2017 | 28.68 (27.65–29.71) |
| Midwest | 2018 | 28.82 (27.81–29.84) |
| Midwest | 2019 | 30.22 (29.19–31.24) |
| Midwest | 2020 | 32.58 (31.52–33.63) |
| Midwest | total | 30.64 (30.40–30.88) |
| South | 1999 | 28.95 (27.99–29.91) |
| South | 2000 | 28.63 (27.68–29.58) |
| South | 2001 | 28.27 (27.33–29.21) |
| South | 2002 | 28.54 (27.60–29.48) |
| South | 2003 | 28.00 (27.08–28.93) |
| South | 2004 | 27.60 (26.68–28.51) |
| South | 2005 | 27.17 (26.27–28.06) |
| South | 2006 | 28.32 (27.42–29.23) |
| South | 2007 | 27.50 (26.62–28.39) |
| South | 2008 | 26.97 (26.10–27.83) |
| South | 2009 | 26.21 (25.37–27.05) |
| South | 2010 | 27.38 (26.52–28.23) |
| South | 2011 | 26.65 (25.82–27.48) |
| South | 2012 | 26.93 (26.11–27.75) |
| South | 2013 | 27.49 (26.68–28.31) |
| South | 2014 | 26.56 (25.77–27.35) |
| South | 2015 | 25.12 (24.37–25.88) |
| South | 2016 | 25.64 (24.89–26.39) |
| South | 2017 | 26.92 (26.16–27.67) |
| South | 2018 | 28.06 (27.30–28.82) |
| South | 2019 | 28.76 (28.00–29.51) |
| South | 2020 | 31.73 (30.94–32.51) |
| South | total | 27.64 (27.46–27.82) |
| West | 1999 | 31.10 (29.76–32.43) |
| West | 2000 | 30.05 (28.75–31.36) |
| West | 2001 | 28.70 (27.45–29.96) |
| West | 2002 | 29.49 (28.23–30.76) |
| West | 2003 | 28.55 (27.32–29.78) |
| West | 2004 | 29.17 (27.94–30.40) |
| West | 2005 | 27.59 (26.40–28.77) |
| West | 2006 | 29.05 (27.85–30.25) |
| West | 2007 | 27.73 (26.57–28.89) |
| West | 2008 | 28.70 (27.53–29.86) |
| West | 2009 | 29.12 (27.96–30.28) |
| West | 2010 | 31.07 (29.89–32.26) |
| West | 2011 | 28.78 (27.66–29.90) |
| West | 2012 | 29.01 (27.90–30.11) |
| West | 2013 | 27.43 (26.37–28.49) |
| West | 2014 | 28.10 (27.04–29.16) |
| West | 2015 | 26.83 (25.81–27.84) |
| West | 2016 | 26.43 (25.44–27.42) |
| West | 2017 | 28.22 (27.21–29.23) |
| West | 2018 | 27.39 (26.42–28.37) |
| West | 2019 | 27.96 (26.99–28.94) |
| West | 2020 | 30.60 (29.60–31.60) |
| West | total | 28.62 (28.38–28.85) |
